# Evaluation of a combined MxA and CRP point-of-care immunoassay to identify viral and/or bacterial immune response in patients with acute febrile respiratory infection

**DOI:** 10.3402/ecrj.v2.28245

**Published:** 2015-12-10

**Authors:** Robert Sambursky, Nathan Shapiro

**Affiliations:** 1 RPS Diagnostics, Sarasota, FL, USA; 2 Beth Israel Deaconess Medical Center, Boston, MA, USA

**Keywords:** respiratory infection, immunoassay, immune response, virus, bacteria, CRP, MxA, point-of-care

## Abstract

**Background:**

Challenges in the clinical differentiation of viral and/or bacterial respiratory infection lead to the misappropriation of antibiotics and increased healthcare costs. A tool to facilitate rapid and accurate point-of-care (POC) differentiation is needed.

**Methods and findings:**

A prospective, single center, blinded, observational clinical trial was conducted at Beth Israel Deaconess Medical Center from December 2012 to August 2013 to determine the accuracy of a POC immunoassay to identify a clinically significant immune response to viral and/or bacterial infection. Sixty patients with acute febrile respiratory infection (19 pharyngitis and 41 lower respiratory tract infection [LRTI]) were enrolled. Participants provided fingerstick blood for immunoassay testing (myxovirus A [MxA] and c-reactive protein [CRP]) and four oropharyngeal samples for viral PCR and routine bacterial cell culture. A venous blood sample was collected. An ELISA was used to measure CRP and MxA. Paired serological testing was used to confirm atypical bacteria. A urine sample was provided for *Streptococcus* and *Legionella* antigen testing. Patients with suspected LRTI had sputum and blood cultures, chest X-ray, and WBC count measured. Viral infection was confirmed if oropharyngeal PCR was positive for viral pathogens. Bacterial infection was confirmed in positive throat or sputum cultures. Elevated immunoglobulin M antibodies or twofold increase in IgG antibodies between acute and convalescent phase indicated atypical bacteria. Positive *Streptococcus* or *Legionella* urine antigen assays also confirmed bacterial infection. The immunoassay correctly categorized subjects as 92% (22/24) negative, 80% (16/20) with bacterial infection, and 70% (7/10) with viral infection.

**Conclusions:**

The interplay between an MxA value and a semi-quantitative CRP value can aid in the differentiation of infectious etiology. In isolation, neither MxA nor CRP alone is sensitive or specific. However, the pattern of results in a rapid immunoassay provides a sensitive and specific method to differentiate acute febrile respiratory infections. This diagnostic information may help reduce antibiotic misuse and resistance and lower healthcare costs.

Viral and bacterial respiratory infections represent a major source of morbidity, mortality, and healthcare costs. According to the Center for Disease Control and Prevention (CDC), antimicrobial resistance is one of the most serious health threats facing the United States. Approximately 80% of all antimicrobials are prescribed in primary care, and up to 80% of these are for respiratory tract indications (1). In many of these indications, antibiotics are unlikely to provide clinical benefit to patients (1, 2). The majority of antibiotics prescribed to adults in ambulatory practice in the United States can be attributed to the lack of clinically differentiating features (3, 4). Although untreated bacterial infections may cause serious complications, treating viral illnesses or non-infective causes of inflammation with antibiotics is not only ineffective but also contributes to the development of resistance, increased costs, and the risk of antibiotic allergy and/or toxicity (5). Antibiotic-resistant infections add nearly $20 billion in excess direct healthcare costs in the United States, not including additional costs to society for lost productivity that may be as high as $35 billion a year. The ideal infectious disease biomarker would impact this health threat through the combination of diagnostic, prognostic, and therapeutic follow-up characteristics (6).

C-reactive protein (CRP) is an acute-phase protein with normal serum concentrations of less than 3 mg/L (7) that increases during an inflammatory process, especially following severe infection (8). In the presence of severe infection or inflammation, CRP can rise above 500 mg/L (9). Bacterial infection is a potent stimulus of marked CRP elevation (10), which occurs within 4–6 h of infection and peaks after 36 h (7). Following antibiotic treatment, CRP levels fall rapidly (11, 12). The rise in CRP usually corresponds with the extent of bacterial infection (13). A normal CRP level in a patient with symptoms of respiratory infection most likely indicates a non-invasive, self-limiting infection that does not require referral to hospital or antibiotic treatment (14–16).

Serum CRP is not usually elevated above 10 mg/L in viral infection (17); however, invasive adenovirus and influenza can raise CRP to 10–80 mg/L (18). A meta-analysis of 10 studies that looked at a single value for serum CRP to be used as a cutoff for bacterial disease, including both gram-positive and gram-negative bacteria, resulted in a bimodal outcome, with three studies recommending that the CRP cutoff value be set between 6 and 20 mg/L and seven studies recommending a cutoff of 60–100 mg/L (19).

Myxovirus resistance protein A (MxA) is a derivative of interferon alpha/beta cells that becomes elevated in the presence of viral infections, but it is not specific to a particular type of virus. MxA has a low basal concentration of less than 50 ng/mL, a fast induction time of 1–2 h after infection, and a long half-life of 2.3 days (20, 21). MxA peaks at 16 h and remains elevated in the presence of elevated interferon (22). MxA protein expression in peripheral blood has been shown to be a sensitive and specific marker for viral infection (17, 23–27). Viral infections elevate MxA levels while only having a modest increase in CRP levels (10, 28–30).

In isolation, neither MxA nor CRP alone is sensitive or specific at identifying both viral and/or bacterial infection. Reviewing several systematic analyses of CRP shows that low cutoff values of CRP demonstrate high sensitivity and low specificity for detecting bacterial infection, whereas high cutoff values of CRP show low sensitivity and high specificity for detecting bacterial infection (6, 9, 19, 31). MxA specifically identifies viral infection; it is not sensitive for bacterial infection. Thus, by simultaneously examining two levels of CRP (≥20 mg/L serum equivalent and ≥65 mg/L serum equivalent), each in combination with the presence of elevated levels of MxA (≥40 ng/ml), it is possible to enhance the sensitivity and specificity of the CRP marker. The interplay between a semi-quantitative value for CRP and MxA can help to identify patients with a clinically significant underlying immune response consistent with a suspected respiratory infection from those patients representing a microbiologically unconfirmed respiratory illness (MURI). These markers will also simultaneously aid in the differentiation of viral and bacterial acute febrile respiratory infections. Examined together in a 15-min point-of-care (POC) test, these markers provide a sensitive and specific means to assess clinical significance and differentiate acute febrile respiratory infections.

## Material and methods

A prospective, single center, blinded, clinical feasibility trial was performed at Beth Israel Deaconess Medical Center (a Harvard Medical School teaching tertiary care hospital) from December 2012 to August 2013. The feasibility trial planned an estimated minimum sample size enrollment of 20 negative patients, 10 confirmed viral patients, and 10 confirmed bacterial patients. The study enrolled 60 patients (34 males and 26 females) with an acute febrile respiratory infection. All subjects older than 17 years of age who presented with fever with acute respiratory symptoms consistent with infection, or reported having a body temperature of ≥100.5°F in the last 48 h, were considered eligible for the study (Supplementary file). At enrollment, the 36 case subjects were separated into two groups: 12 with presumed pharyngitis and 24 with presumed LRTI (Supplementary file). If a patient did not have a fever and was asymptomatic (as described in the inclusion criteria), the patient was considered for inclusion as a control subject (Supplementary file). Twenty-four patients were enrolled into the control group.

The study was approved by the Beth Israel Deaconess Medical Center Committee for Clinical Investigations with a written consent. Study personnel collected up to seven samples from all patients including one venous blood sample, four oropharyngeal samples, a urine sample, and a fingerstick blood sample that was applied to a rapid, POC immunoassay, according to the manufacturer's instructions for use. In addition to the preceding tests, subjects suspected of having an LRTI had sputum and blood cultures as well as a chest X-ray. Participation of subjects required one visit with one follow-up visit. The follow-up visit, 4–6 weeks after the initial visit, was necessary to collect a venous blood sample for follow-up serology testing.

**Fig. 1:**
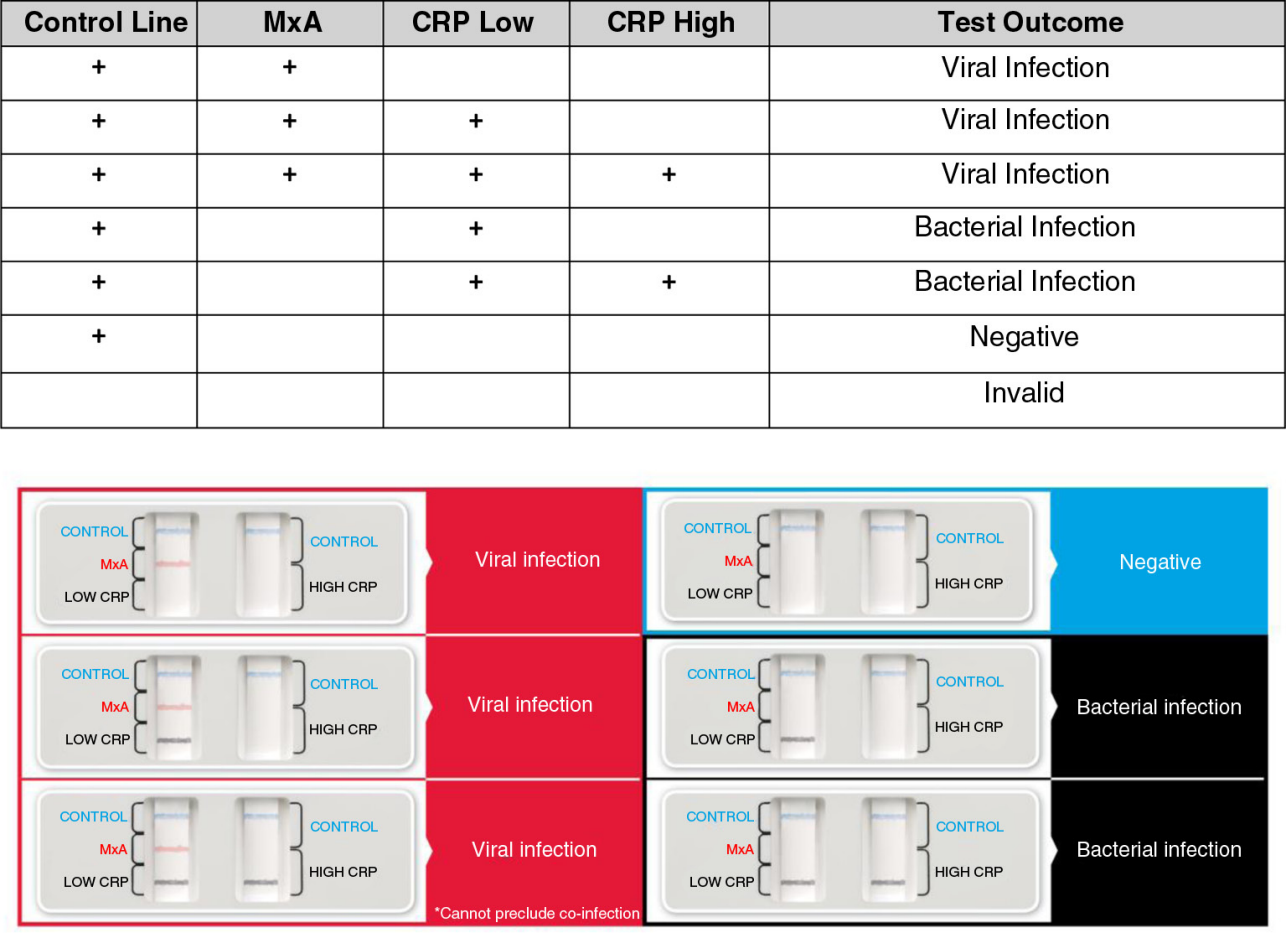
Immunoassay interpretation.

The immunoassay was interpreted by identifying the presence of the control lines or result lines according to Fig. 1. Two of the oropharyngeal samples were sent for a viral respiratory PCR panel (Luminex xTAG, Austin, TX) and other viral PCR testing, whereas the other two oropharyngeal samples were sent for routine bacterial cell culture. A 5 mL peripheral venous blood sample, collected in a purple top tube (ethylenediaminetetraacetic acid [EDTA]), was sent for quantitative MxA enzyme–linked immunosorbent assays (ELISA) testing using the MxA Protein ELISA Test Kit (Kyowa Medex Co., Ltd., Tokyo, Japan) and WBC measurement. A second sample, collected in a red top tube, was used for CRP testing with the High Sensitivity CRP Enzyme Immunoassay Test Kit (Biocheck, Inc., Foster City, CA).

Diagnosis of *Chlamydia pneumoniae* and *Mycoplasma pneumoniae* was determined by PCR and performed by means of paired serology at the time of enrollment and at 4–6 weeks thereafter. Commercially available ELISA tests (Ani Labsystems Ltd. Oy., Vantaa, Finland) were used according to the manufacturer's instructions for the detection of immunoglobulin M (IgM) and IgG antibodies to *M. pneumoniae* and *C. pneumoniae*. Atypical bacterial infection was confirmed if *M. pneumoniae* and *C. pneumoniae* were identified by PCR, *M. pneumoniae* and *C. pneumoniae* IgM antibodies were detected, or a twofold increase in IgG antibodies between acute and convalescent phase samples was found.

A definitive typical bacterial infection was considered when a bacterium was cultured from blood, sputum, or if the urine antigen assay for *Legionella* or *Streptococcus* was found to be positive. All subjects suspected of an LRTI had peripheral venous blood collected and sent for plating on routine bacterial blood cultures. Upon reaching the clinical laboratory, the specimens were divided into samples for plating on blood and chocolate agar. All specimens were processed within 24 h of collection, and a single colony-forming unit (CFU)/mL of a single bacterial species indicated an infection and not colonization.

Expectorated sputum was collected from subjects with a productive cough and a presumptive LRTI. Only samples that had more than 25 polymorphonuclear leukocytes and less than 25 squamous cells per microscope high-power field were plated for culture (32). The quality of sputum samples was evaluated by assessing the number of inflammatory and epithelial cells. A definitive bacterial infection was considered when any Group A beta hemolytic strep growth occurred or any other bacterial growth greater than 1×10^5^ CFU/mL occurred in oropharyngeal samples or sputum samples.

Urine samples were collected and assayed for *Streptococcal pneumoniae* and *Legionella pneumophila* antigen. Immunochromatographic membrane tests (Alere BinaxNOW *S. pneumoniae* and BinaxNOW *Legionella*, Waltham, MA) were performed on urine samples for detection of *S. pneumoniae* and *L. pneumophila* antigens. Identification of *L. pneumophila* by PCR also confirmed the diagnosis of *Legionella*.

Definitive viral infection was confirmed if the oropharyngeal PCR respiratory panel (Luminex xTAG; Austin, TX) or other viral PCR was positive for viral nucleic acid.

**Fig. 2:**
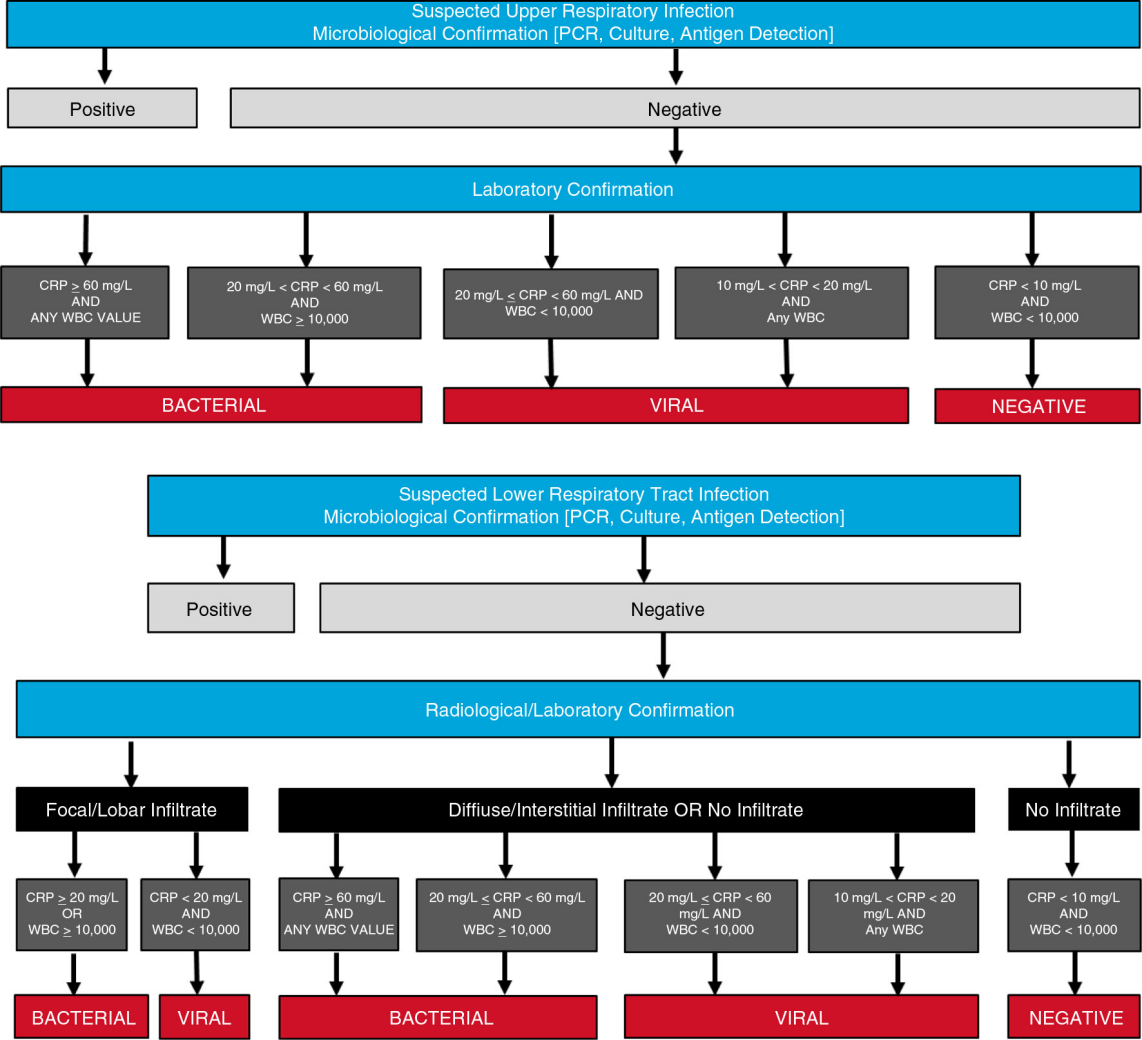
Clinical diagnostic algorithm.

Subjects who did not have a definitive microbiological confirmation of disease were characterized according to an algorithm incorporating additional radiological and laboratory findings (Fig. 2). The presence of radiologic evidence of diffuse infiltrates by chest X-ray suggested a viral infection while the presence of radiologic evidence of a focal lobar process or infiltrate by chest X-ray suggested a bacterial infection. In addition, significantly elevated WBC count over 12,000 mm^3^ was interpreted to suggest bacterial infection.

## Results

Two invalid tests occurred and four subjects were diagnostically indeterminant because of specimen leakage or rejection. Of the remaining 54 patients, the immunoassay correctly identified 92% (22/24) of subjects as negative for infection (95% CI: 74.2–97.7), 80% (16/20) of confirmed bacterial infections (95% CI: 56.3–94.1), and 70% (7/10) of confirmed viral infections (95% CI: 39.7–89.2). The percent negative and positive agreement of the test was calculated according to the charts in Table 1.

**Table 1:** Immunoassay performance results

Comparator (microbiological, radiological, laboratory assessment)					
Pharyngitis (*N*=16)					
Immunoassay		Bacterial	Viral	Negative	% Correct
	Bacterial	4	1	0	80% (4/5)
	Viral	1	3	0	75% (3/4)
	Negative	0	0	7	100% (7/7)
Total		5	4	7	
LRTI (*N*=38)					
Immunoassay		Bacterial	Viral	Negative	% Correct
	Bacterial	12	1	2	80% (12/15)
	Viral	1	4	1	67% (4/6)
	Negative	2	1	14	82% (14/17)
Total		15	6	17	38
Pharyngitis and LRTI combined (*N*=54)					
Immunoassay		Bacterial	Viral	Negative	% Correct
	Bacterial	16	2	2	80% (16/20)
	Viral	2	7	1	70% (7/10)
	Negative	2	1	21	92% (22/24)
Total		20	10	24	54

Of the 41 enrolled patients with LRTI, 26 were males and 15 were females with an age range of 22–89 and a mean age of 51 years. Of the 19 patients enrolled with pharyngitis, 8 were males and 11 were females with an age range of 18–69 and a mean age of 37 years. Viral pathogens detected by PCR included influenza A, influenza B, parainfluenza 2, parainfluenza 3, and HSV-1. Three asymptomatic controls had rhinovirus detected without an associated elevation in MxA; this was deemed likely colonization and was excluded from the microbiological confirmation. If both a viral and bacterial infection were confirmed microbiologically, this was characterized as a bacterial infection.

## Discussion

Acute febrile respiratory infections frequently have no confirmed etiology, both for URI, such as pharyngitis, and LRTI, such as community-acquired pneumonia (CAP), despite an extensive combination of microbiological and molecular diagnostic techniques, including molecular testing for both bacterial and viral pathogens. A review of the recent scientific literature revealed numerous prospective clinical studies evaluating the etiology of acute respiratory infections and reporting a failure of pathogen detection for 24–44% of the patients (33–39). In the present study, 44% (24/54) of patients had no microbial confirmation of infection. Patients without a microbial confirmation and a limited immune response may represent a potentially less significant clinical case of MURI.

A rapid, differentiating POC test has profound potential healthcare implications since distinguishing viral from bacterial infections in the clinical setting has been shown to be challenging, especially early in the disease process (3, 4). Van Gageldonk-Lafeber et al. observed no association between detected bacterial and viral pathogens and either diagnosis made by general practitioners or subject's reported symptoms (40). Moreover, physical examination alone was shown to have a sensitivity of 50–70% and specificity of 60–75% as well as a negative predictive value of 60% and a positive predictive value of 50% (41, 42). The difficulty in establishing an etiologic outpatient diagnosis in acute febrile respiratory illness stems from overlap in symptoms and signs, limitations of available diagnostic tests, and the time lag in receiving results from laboratory tests.

According to Korppi, CRP measurement is recommended as the first-line method of screening suspected bacterial inflammation (43). Several studies have indicated that CRP is feasible and accurate at differentiating pneumonia from acute bronchitis (6, 44–46). Pneumonia is associated with elevated serum CRP levels greater than 10 mg/L, whereas severe pneumonia has serum CRP typically greater than 100 mg/L (11, 47). In Scandinavia, POC CRP testing is part of the routine evaluation of patients with LRTI, and its use has proved cost-effective (48, 49).

Both CRP and procalcitonin (PCT) concentrations have been used to initiate and monitor antibiotic use for LRTI (50, 51). PCT has been suggested (52) for monitoring community-acquired outpatient infections as a POC test; however, the relatively high cost makes it less desirable for high-incidence infections in family practice (53). In general, the specificity of single biomarkers in terms of etiologic distinction between bacterial and viral infections remains a problem (19, 54). Both CRP and PCT as a single biomarker are more specific for bacterial etiology of infection at high concentrations, but lower concentrations of CRP and PCT are often observed during both viral and bacterial infections (54, 55). The combination of either PCT or CRP alongside MxA would be helpful in differentiating viral from bacterial infection. Attempts at panel tests, including CRP combined with IL-18 because of its role in anti-viral immunity, have been unsuccessful in differentiating viral from bacterial infection (55).

Engelmann et al. prospectively examined 553 children consisting of 44 uninfected controls and 77 confirmed viral infections and found that MxA levels in the uninfected control group were significantly lower than in the patients with confirmed viral infection (P<.0001). This study also found that MxA levels were significantly lower in patients with confirmed bacterial infection than in patients with confirmed viral infection (P<.0001) and that CRP levels were higher in patients with confirmed bacterial infection than in those with confirmed viral infection (P<.0001). Using a combination of MxA and CRP as indicators of infection, the ROC curve, sensitivity, and specificity for differentiating between bacterial and viral infections was 0.84 (95% CI: 0.75–0.94), 80.9% (95% CI: 66.7–90.8), and 87.9% (95% CI: 71.8–96.5), respectively (56).

Higher MxA levels in patients with viral infection compared with patients with bacterial infection can be explained by the fact that MxA protein is induced exclusively by type 1 IFN and not by IFN-gamma, IL-1, TNF-alpha, or any of the other cytokines induced by bacterial infection (57). Serum type 1 IFN levels remain within normal limits, even in patients with severe bacterial infections (58, 59). There is substantive data that demonstrate that human infection with respiratory syncytial virus (RSV), influenza A and B, adenovirus, parainfluenza virus, and metapneumovirus stimulate a robust cytokine response that includes gamma interferon (60, 61), whereas rhinovirus does not typically stimulate a significant elevation MxA (62). The magnitude of the IFN response varies with the type of the inciting virus (60). Moreover, a deficiency in the receptor for IFN is reported to increase the severity of respiratory viral infection (63). MxA has been found to be elevated in common respiratory viral infections as well as common viral gastrointestinal infections (Table 2) (17, 25–27, 64, 65).

**Table 2:** A summary of clinical studies testing for MxA

Study	Type of study	Sample size	Clinical syndrome	Matrix	Subject ages	Viruses associated with elevated MxA
Forster et al. (25)	Prospective	*N*=182; 81 viral infections; 101 normal controls and non-viral respiratory illness	Respiratory illness	182 nasopharyngeal and 92 whole blood	Infants and children	RSV, adenovirus, influenza B, parainfluenza 2, parainfluenza 3, rotavirus
Halminen et al. (26)	Prospective	*N*=133; 28 viral infections; 12 bacterial infections; 80 normal controls	Respiratory and GI illness	Whole blood	Children with infections; adult and children healthy controls	RSV, adenovirus, rotavirus, influenza, herpes simplex virus, Epstein–Barr virus
Chieux et al. (27)	Prospective	*N*=75; 32 viral infections; 13 bacterial infections; 30 normal controls	Respiratory and GI illness	Whole blood	Children with infections; adult and children healthy controls	RSV, adenovirus, rotavirus, CMV
Chieux et al. (65)	Prospective	*N*=69; 43 viral infections; six bacterial infections; 20 normal controls	Respiratory illness	Whole blood	Infants	RSV, adenovirus, rotavirus, CMV
Nakabayashi et al. (17)	Prospective	*N*=174; 122 children with acute fever; 52 normal controls	Respiratory and GI illness	Whole blood	Infants and children	RSV, adenovirus, rotavirus, CMV, influenza, varicella
Kawamura et al. (64)	Prospective	*N*=60; 42 children with acute fever; 18 normal controls	Respiratory and GI illness	Whole blood	Infants and children	RSV, adenovirus, rotavirus, influenza

The high sensitivity of PCR allows the detection of minimal amounts of viral nucleic acids, but the clinical relevance of positive test results is not clear because small amounts of a respiratory virus could represent asymptomatic colonization or postinfectious shedding (66). When asymptomatic control patients are compared with patients with respiratory illnesses, PCR detects the presence of viruses in 19–44% of the control patients, suggesting transient colonization or persistence, most commonly associated with rhinovirus and coronavirus (40, 66–69). Nokso-Koivisto showed that 81% of the children with virus-positive samples had previous respiratory symptoms or had family members with concurrent respiratory symptoms (70). However, viruses such as influenza, parainfluenza, metapneumovirus, and RSV are rarely detected in asymptomatic subjects, and when present, they suggest active infection (67, 71, 72). Since these viruses all seem to be rapidly cleared from the respiratory tract after an infection, PCR is a suitable diagnostic method for determining their infection (73).

Rhinovirus is considered a relatively mild pathogen that can colonize the nasal mucosa without causing symptoms (69). Rhinovirus and coronavirus cause common cold and do not typically cause an invasive infection or fever in immunocompetent hosts, or stimulate IFN or MxA (70, 74). This suggests that a causal inference based on the detection of these viruses in symptomatic patients should be made with caution. An accompanying immune response with elevated MxA is suggestive of a true rhinoviral or coronaviral infection while CRP elevation in the absence of elevated MxA suggests a bacterial infection.

This study has several limitations. First, the sample size is small, especially for the viral infection group. Second, there children under the age of 17 were not enrolled. In addition, in this study, when a bacterial infection was confirmed by the comparator method, it was deemed a bacterial infection regardless of the presence of any viral co-infection. There is no universally accepted method for determining colonization from active invasive infection or a method for clinically categorizing symptomatic patients without definitive microbiological confirmation of disease. Finally, there is some element of tautology in this study because CRP was used as part of the comparative method. Alternatively, future studies could consider the use of PCT as a surrogate marker for bacterial infection. Despite these limitations, the interplay between a semi-quantitative value for CRP and MxA appears to aid in the identification and differentiation of infectious etiology and warrants further investigation.

## Conclusions

The overuse of antibiotics is the most important factor leading to antibiotic resistance; however, at least 50% of all antibiotics are prescribed for acute febrile respiratory infections and are not needed or are not optimally effective as prescribed (5). Unnecessary overuse of antibiotics is associated with increased antibiotic resistance for common bacteria, adverse reactions, and high costs for the entire healthcare system, including providers, payers, and patients.

Viral and bacterial infections are contagious and difficult to clinically differentiate because of a significant overlap in signs and symptoms. Difficulty in obtaining relevant specimens, the low sensitivity or specificity of the tests used, high costs, and the absence of test results within the critical window for initiating adequate treatment often result in the prescription of antibiotic therapy in the absence of a bacterial infection. Empiric treatment is especially prevalent in young children who cannot verbalize their symptoms and in the outpatient setting where access to laboratory diagnostics is limited. Independently, antigen testing, cell culture, and PCR cannot differentiate active invasive infection from bacterial or viral colonization. However, microbiological confirmation in the presence of an elevated host immune response is more indicative of a clinically significant infection requiring therapeutic intervention.

In an immune-competent person, PCT and CRP are ideal biomarkers for determining a host response consistent with a bacterial confirmation while MxA serves to confirm the presence of a significant viral infection. In isolation, neither MxA nor CRP alone is sensitive or specific in identifying viral and/or bacterial infection. However, a multiplexed pattern of results consisting of the combined interpretation of low CRP, high CRP, and elevated levels of MxA may provide a sensitive and specific way to identify an immune response to a viral and/or bacterial infection. Use of a rapid test may result in lesser instances of unnecessary antibiotic use, reduce antibiotic resistance, and lower healthcare costs.

## Supplementary Material

Evaluation of a combined MxA and CRP point-of-care immunoassay to identify viral and/or bacterial immune response in patients with acute febrile respiratory infection
