# Supplementary material for: Evaluation of a combined MxA and CRP point-of-care immunoassay to identify viral and/or bacterial immune response in patients with acute febrile respiratory infection
Source: Eur Clin Respir J. 2015 Dec 10;2:10.3402/ecrj.v2.28245. doi: 10.3402/ecrj.v2.28245 (PMC4676840; doi:10.3402/ecrj.v2.28245)
Supplement: Evaluation of a combined MxA and CRP point-of-care immunoassay to identify viral and/or bacterial immune response in patients with acute febrile respiratory infection [file ECRJ-2-28245-s001.pdf]

Supplemental Material: Inclusion/Exclusion Criteria

<u>Inclusion Criteria</u>

General inclusion criteria for the acute respiratory infection patients
All subjects older than 17 years of age who presented with acute respiratory symptoms consistent with a respiratory infection that had a fever, or reported having had a fever in the last 48 hours, greater than or equal to 100.5�F, were considered eligible for the study.

Additional inclusion criteria for suspected upper respiratory tract infection (URTI)
Presence of a sore throat and at least 1 clinical sign listed below:

<u>Signs</u>
1. Swollen or tender anterior cervical nodes
2. Tonsillar erythema
3. Tonsillar swelling
4. Tonsillar exudate

Additional inclusion criteria for suspected lower respiratory tract infection (LRTI)
A) ≥ 2 symptoms below or the presence of at least 1 symptom and 1 sign listed below:

<u>Symptoms</u>
- Cough
- Chills
- Dyspnea
- Fatigue
- Purulent sputum
- Pleuritic pain

<u>Signs</u>
- Tachypnea (respiratory rate >20/min)
- Tachycardia (heart rate >100/min)
- SAO2 < 95%
- Abnormal auscultation: crackles or rales

B) Subjects 17 years of age or older that present with a suspected LRTI were enrolled in the clinical study. Subjects must present with a fever greater than or equal to 100.5�F

C) Radiologic evidence of a lobar process or diffuse infiltrates

Inclusion criteria for case LRTI group (criteria A, B, and C <u>all</u> had to be met for study inclusion)
A) ≥ 2 symptoms below <u>or</u> the presence of at least 1 symptom and 1 sign listed below:

<u>Symptoms</u>
- Cough

- Chills
- Dyspnea
- Fatigue
- Purulent sputum
- Pleuritic pain

<u>Signs</u>

- Tachypnea (respiratory rate >20/min)
- Tachycardia (heart rate >100/min)
- SAO2 < 95%
- Abnormal auscultation: crackles or rales

B) Subjects older than 17 years of age who present with a suspected lower respiratory tract illness were enrolled in the clinical study. Subjects must present with a fever greater than or equal to 100.5° F (oral or tympanic) for $\leq$ 7 days

C) Radiologic evidence of focal (lobar) or diffuse (interstitial) infiltrates

Inclusion criteria for control group

- Subjects older than 17 years of age who present during a wellness visit
- Subjects must be asymptomatic for a systemic or local infection

Exclusion Criteria

Exclusion criteria for case group

- Taking immunosuppressive or chemotherapeutic medications 30 days prior to study entry
- Taking antibiotics or antiviral therapy 30 days prior to study entry
- Receiving interferon therapy (eg. multiple sclerosis, HIV, HBV, HCV) in the last 30 days
- Received a live viral immunization in the last 30 days

Exclusion criteria for control group

- Fever $\geq$ 100.5°F (oral or tympanic) in last 14 days
- Nasal congestion
- Rhinorrhea
- Sore throat
- Headache
- Myalgia
- Chills
- Skin lesions
- BMI $\geq$ 50 kg/m^2
- Active pulmonary disease or a significant cough
- Autoimmune or rheumatologic disease

- History of cancer
- History of a myocardial infarction or stroke in the last 30 days
- Chronic bacterial infection or osteomyelitis
- Taking immunosuppressive or chemotherapeutic medications in the last 30 days prior to study entry
- Known chronic viral infections such as HIV, HCV, HBV, or CMV
- Active tuberculosis
- Taking antibiotics or antiviral therapy in the last 30 days prior to study entry
- History of significant trauma or burns (> 5% total body surface area or full thickness [3rd°]) in the last 90 days
- Acute or chronic (greater than 30 days) diarrhea and/or vomiting
- Urinary tract symptoms in the last 14 days
- Active skin, ocular, or neurologic infections
- Major surgery (requiring intravenous anesthesia and/or respiratory assistance) in the last 90 days
- History of multiple sclerosis
- Received interferon therapy (eg. Multiple Sclerosis, HIV, HBV, HCV) in last 30 days
- Received a live viral immunization in the last 30 days
